# Generation of functional organs from stem cells

**DOI:** 10.1186/2045-9769-2-1

**Published:** 2013-01-22

**Authors:** Yunying Liu, Ru Yang, Zuping He, Wei-Qiang Gao

**Affiliations:** 1_9 State Key Laboratory of Oncogenes and Related Genes, Stem Cell Research Center, Renji Hospital, Shanghai Jiaotong University School of Medicine, Shanghai, China; 2_9 Med-X Research Institute, Shanghai Jiaotong University, Shanghai, 200127 China

**Keywords:** Stem cells, Functional organs, Blastocyst complementation, Decellularization, Recellularization, Tissue engineering

## Abstract

We are now well entering the exciting era of stem cells. Potential stem cell therapy holds great promise for the treatment of many diseases such as stroke, traumatic brain injury, Alzheimer’s disease, Parkinson’s disease, amyotrophic lateral-sclerosis, myocardial infarction, muscular dystrophy, diabetes, and etc. It is generally believed that transplantation of specific stem cells into the injured tissue to replace the lost cells is an effective way to repair the tissue. In fact, organ transplantation has been successfully practiced in clinics for liver or kidney failure. However, the severe shortage of donor organs has been a major obstacle for the expansion of organ transplantation programs. Toward that direction, generation of transplantable organs using stem cells is a desirable approach for organ replacement and would be of great interest for both basic and clinical scientists. Here we review recent progress in the field of organ generation using various methods including single adult tissue stem cells, a blastocyst complementation system, tissue decellularization/recellularization and a combination of stem cells and tissue engineering.

## Introduction

Stem cells are undifferentiated cells found in the body which have the ability to continuously divide, self-renew themselves and differentiate into various kinds of cells. With the capability of self-renewal, pluripotency and differentiation, stem cells have been believed to be useful for treatment of a wide variety of diseases in the future, including stroke, traumatic brain injury, Alzheimer’s disease, Parkinson’s disease, spinal cord injury, baldness, blindness, deafness, wound healing, amyotrophic lateral-sclerosis, myocardial infarction, muscular dystrophy, osteoarthritis rheumatoid arthritis, Crohn’s disease, and diabetes. Amongst the applications, a number of adult stem cell therapies have already been practiced clinically. As an example, hematopoietic stem cell transplantation has been successfully applied to treat leukemia.

In addition to cell replacement therapy using stem cells, organ transplantation has been successfully practiced in clinics for organ failure of the liver or kidney. However, the severe shortage of donor organs has become the main obstacle to expand the organ transplant program. Generation of biological or semi-biological organs could be an alternative approach to solve the problem of the donor organ shortage. Notably, researchers have been hunting for ways to establish a whole organ using stem cells.

In recent years encouraging approaches for functional organ generation have emerged. The present manuscript provides an overview of organ generation using a single adult tissue stem cell, a blastocyst complementation system coupled with a specific stem cell niche, a method of decellularization and recellularization of bio-scaffold, and a combinatorial approach of tissue engineering and stem cells.

### Generation of a functional organ from a single adult tissue stem cell

To demonstrate whether there are true stem cells in a given tissue, one needs to show that a single stem cell purified from the tissue has the capability of generating the entire organ. Up to present, it has been elegantly shown by independent groups that the mammary gland and the prostate can be generated in vivo from a single adult tissue stem cell [1–3].

Two laboratories have reported independently that single stem cells isolated from adult mouse mammary glands are able to produce secretory mammary glands when they are transplanted in the fat-pad in mice [1, 2]. It is long believed that there are stem cells in the mammary glands because this organ has the capability of undergoing an extensive growth at puberty and a second phase of expansion and retraction during pregnancy under the regulation of estrogen [4]. However, due to the lack of defined markers, there has been no reliable method to isolate mammary stem cells. This hypothesis was not proven until the work by the two groups. Based on some previous work by Clark and colleague [5], Shackleton et al. isolated putative mouse mammary stem cells using specific cell-surface markers (Lin^-^, CD29^hi^, CD24^+^) by FACS. They demonstrated these Lin^-^CD29^hi^CD24^+^ mammary cells have *in vitro* sphere formation capability and the ability to repopulate all mammary epithelial cells after transplantation into the fat-pad. Importantly, the investigators employed a lineage-tracer in the stem cells so to follow their ultimate phenotypes *in vivo*. They showed elegantly that a single cell within the Lin^-^CD29^hi^CD24^+^ population of the mouse mammary gland, marked with a LacZ report transgene, can reconstitute a completely functional mammary gland *in vivo*. Notably, the transplanted cell contributes to both the luminal and myoepithelial lineages and generates milk-producing lobuloalveolar units during pregnancy. This is the first report to demonstrate that a single adult tissue stem cell has multi-lineage differentiation capacity to produce a functional organ in an *in vivo* setting.

Similarly, using a colony-formation *in vivo* assay and an *in vivo* renal capsule transplantation approach, Gao and his colleagues have also reported that a single stem cell isolated from the adult mouse prostate epithelium has the capacity to generate a functional prostate [3]. Actually, prostate stem cells were postulated to exist because of its regenerative feature following androgen deprivation and replacement more than twenty years ago [6]. Because cell death mainly occurs in the luminal cell compartment after castration and cell proliferation mainly happens in the basal compartment following androgen replacement, stem cells are generally believed to reside in basal cell compartment and are able to repopulate the entire prostate epithelial cells.

First, this group identified a new marker for prostate stem cells, CD117, based on the following 6 features: 1) It is enriched in proximal region of the prostate; 2) It is mainly expressed in basal cell population; 3) It is upregulated after castration and returned to normal levels following androgen replacement; 4) Only CD117^+^ cells, but not the CD117^-^ cells, can form colony structures with lumen *in vitro*; 5) It can generate prostate epithelial glandular structures *in vivo*; 6) CD117+ cells from 2^nd^ and 3^rd^ generation grafts also have the self-renewal capability.

To demonstrate whether a single stem cell has the ability to generate a prostate structure, the investigators first further enriched the stem cell population by sorting the cells expressing multiple stem cell markers, Sca-1^+^CD133^+^CD44^+^CD117^+^, into individual wells of a 96-well plate, verified them under a microscope, and transplanted them in a combination with rat embryonic urogenital sinus mesenchymal cells (rUGM) under the renal capsule of athymic nu/nu mouse hosts. Three months later, they removed the kidneys and analyzed the fate of the grafted cells. Of the 97 single-cell transplants, there were about 1/7 Sca-1^+^CD133^+^CD44^+^CD117^+^ grafts that demonstrate a branching pattern with epithelial tubules composed of prostatic basal, luminal and neuroendocrine lineages by the histological and immunocytochemical examination.

The generation of mammary gland and prostate acini from single stem cell implants in mice is a major breakthrough. These findings raise the possibility that people who have lost their mammary glands or prostates due to cancer could grow new ones. On the other hand, it is believed that in some tissues, there are cancer stem cells that are tumor-initiating cells. Cancer stem cells might be derived from normal stem cells in which specific tumor suppressor genes are mutated or lost [7]. Therefore, although the prostate glands were grown in mice but the research may aid the identification of markers for cancer stem cells, which may help diagnosis and more efficient treatment of prostate cancer in humans. These studies on reconstruction of mammary and prostate glands using single adult stem cells have important ramifications not only for tissue repair/regeneration, but also for identification of mammary and prostate cancer stem cells. In other words, we envision that the potential use of single stem cells in clinic will ultimately change the treatment paradigm for human disorders more than mammary gland injury and prostatic disease.

### Generation of organs using a blastocyst complementation system

In addition to single stem cells prepared from specific adult tissue, embryonic stem cells (ESCs) have been shown to be able to produce specific organs by using a strategy of injection of ESCs from one species into the blastocyst of another species. The development of a specific organ can be precluded by genetic manipulation in the recipient species but still providing a niche for organ development. The pluripotent stem cell-derived cells from the donor species would then developmentally compensate for the defect and produce the missing organ.

The blastocyst complementation system was first reported by Chen et al. by implanting normal mouse embryonic stem cells (mESCs) into the blastocysts derived from Rag2^−/−^ mice to generate T and B lymphocyte lineages [8]. In recent years, this system was applied to generate pluripotent stem cell (PSC)–derived donor organs *in vivo* due to the fact that complex cellular interactions among and within tissues that are required for organogenesis are difficult to recapitulate *in vitro*. For this purpose, PSCs were injected into blastocysts obtained from mutant mice in which the development of a certain organ was precluded by genetic manipulation just to supply as a niche for organ development. The PSC-derived cells would form the missing organ as a result of developmental compensation for the defect.

Kobayashi and colleagues had showed proof-of-principle findings of pancreas generation by injection of PSCs into the blastocysts of pancreas-deficient *Pdx1* ^−/−^ mouse (pancreatogenesis-disabled) [9]. More specifically, they injected induced pluripotent stem cells (iPS) taken from rats into the blastocysts of mice that were unable to grow their own pancreas and were unable to produce insulin. The rat stem cells grew in the special mouse environment in the absent of mouse pancreas. When the mice matured to adulthood, they developed a rat pancreas from the injected rat stem cells. The origin and function of pancreatic cells were analyzed in the PSC-derived rat pancreas in adult mice. Glucose load was tested and the mice showed no signs of diabetes. These findings clearly indicate that functional rat pancreas are successfully generated in *Pdx1* ^−/−^ mice using the inter-species blastocyst complementation.

The same technique has been also used to generate kidney using *Sall1* ^−/−^ mouse blastocysts [10]. Rat ESC or PSC are injected into *Sall1* ^−/−^ mouse blastocysts and the entire rat kidneys are formed by the injected mouse ESC- or PSC-derived cells. These ESC/PSC-derived kidneys connect with ureters and function well. The bladders are full of urine.

Generation of pancreas and kidney via blastocyst complementation is an innovative approach. It demonstrates that this technique can be used to generate complex organ using donor PSCs. With an empty developmental niche, a solid organ will be produced developmentally from PSCs-derived cellular progeny in the vacant space.

The current organ shortage means that patients must endure a long time on waiting lists for transplantation. This blastocyst complementation system may therefore provide a novel approach for organ supply. The ultimate goal of blastocyst complementation system is to generate specific human organs from various pluripotent stem cells. However, such inter-species technology using human stem cells involves ethical issues which has to meet the scientific and ethical guidelines proposed by the International Society for Stem Cell Research (ISSCR) and requires approval from related ethical committees.

### Engineering a whole organ via decellularization of matrix bioscaffolds and recellularization with stem cells

Organ formation requires not only stem cells but also the surrounding stem cell niche or microenvironment and extracellular matrix participation. In fact, three-dimensional tissue scaffold has been demonstrated to be very critical for organ regeneration.

Decellularization and recellularization of matrix bioscaffolds have been shown to be a promising approach for whole-organ tissue engineering in recent years [11]. Donor organs such as the heart [12], liver [13], lung [14], kidney [15] and bladder [16] can be decellularized to an acellular biologic scaffold material and then be recellularized with functional parenchymal cells and/or selected progenitor cell populations. Encouraging results have emerged in animal model studies.

Taylor and colleagues have generated a bioartificial heart which has a heart structure with appropriate cell composition and cardiac pump function using this attractive technique in 2008 [12]. They perfused heart with 1% SDS, a strong ionic detergent, to produce acellular scaffolds, preserved extracellular matrix and original microvascular network, and then repopulated these heart-like constructs with neonatal cardiomyocytes and aortic endothelial cells. When proper physiological stimuli were given, the recellularized organ in the culture was able to further mature and display beating behaviors. Rhythmic contractions were observed by day 4, pump function generated by day 8 and cardiac architecture was formed, as confirmed by histological analysis. The bioartificial heart pumped with an amazing function, which was equivalent to about 2% heart power of an adult or 25% heart function of a 16-week fetus.

Uygun and colleagues modified Taylor’s perfusion decellularization technique to achieve transplantable liver grafts in 2010 [13]. The ischemic livers were perfused through the portal vein with SDS. The intact lobular structure and vascular network were retained in the decellularized liver bioscaffold. The reseeded adult hepatocytes and nonparenchymal cells were able to repopulate to create a recellularized whole liver graft. The graft was transplanted as auxiliary heterotopic graft with portal vein arterializations and animal was shown to be viable for 8 hours after transplantation. The hepatocytes in the recellularized liver graft were metabolically active with functions of urea and albumin secretion. Similarly, Niklason and colleagues regenerated lung grafts using the decellularization and recellularization strategy in 2010 [14]. They removed cellular components from lung matrix, preserved hierarchical branching structures of the airways and vasculature bed in the extracellular scaffold, and reseeded pulmonary and vascular endothelial cells in the acellular lung matrix. The engineered lungs presented pneumonocyte viability and function after implantation, as evidenced by oxygen exchange.

These exciting studies on complex organs, such as the heart, liver and lung as described above, provide insights into the power of the methodology. The technology has multiple advantages. First, a bioartificial organ could theoretically solve the problem of lifelong immunosuppression after organ transplantation. Second, the preserved extracellular matrix and three-dimensional scaffold provide important signals for engraftment of repopulated cells, survival of engrafted cells, and function of newborn organ. Third, the vascular bed in the decellularized bioscaffold allows oxygen and nutrient rapid to be delivered after recellularization and reconnection to the circulation.

The most important concern in the technology is the cell type and the source of cells used for filling the decellularized organ matrix. Different types of cells are needed to reconstruct the parenchyma, vasculature, and support structures underneath. An ideal strategy is that a stem or progenitor cell can self-renew and differentiate into heterogeneous types of cells as needed to form a functional organ. Currently, cell sources are broadly categorized. Stem or progenitor cells used for recellularization include embryonic stem cells (ESCs), fetal cells, adult-derived stem or progenitor cells and adult tissue-derived inducible pluripotent stem cells (iPSCs). Non-stem or progenitor cells used for repopulation are usually parenchymal cells (e.g. cardiomyocytes, hepatocytes, pneumonocytes), vascular cells (e.g. endothelial cells) and supportive cells (e.g. fibroblasts). Whether autologous or allogeneic cells are utilized will depend on cell numbers required and if the need is immediate.

In short, decellularization and recellularization technique is a potential breakthrough in whole-organ tissue engineering. With further development, organs (e.g. heart, liver, lung, kidney, pancreas) can be stripped of their cells and regrow to pump blood, to generate albumin and urea, to exchange oxygen to produce urine and to secret insulin/glucagon with replaced cardiocytes, hepatocytes, alveolar type 2 cells, renal medulla cells and islet α, β cells.

### Generation of organs by a combination of tissue-engineering and seeded stem cells

Tissue-engineering techniques have been used to combine specific stem cells with biocompatible and biodegradable polymer scaffolds to produce a cell polymer implant. The field of tissue engineering has been developed to solve the problem of donor-tissue scarcity either as whole organs or as reconstructive grafts, such as cartilage frameworks for total ear reconstruction [17, 18]. Cao and colleagues were the first to apply polyglycolic acid-polylactic acid to form a polymer template in the shape of a human auricle and seeded chondrocytes into the construct. New tissue-engineered cartilage grew for 12 weeks after implantation under the dorsal skin of mice. The polyglycolic acid-polylactic acid construct degraded gradually following transplantation, but the cartilage was formed and confirmed by morphological and histological analysis [17]. The technique is particularly useful for plastic and reconstructive surgery.

But this system, for quite a long time, seems mainly focused on the exterior instead of the interior of the function of the organ because of the key challenge to integrate fully functioning vascular architectures into the engineered construct. Therefore, the generation of functional human vascular network by Takebe and colleagues provides an innovative strategy to regenerate larger, well-vascularized organs including the liver and seems to solve the major obstacles in regenerating thick, complex tissues. They co-cultured human umbilical vein endothelial cells and mesenchymal stem cells in a matrix made of collagen and fibronectin. The human umbilical vein endothelial cells were labeled with GFP and the human mesenchymal stem cells were with kusabira orange. Vessel-like constructs were formed and detected under a fluorescence microscope *in vitro* seven days after cultivation. More importantly, when the vascular network constructs were implanted into an immunodeficient mouse, they were able to infuse the structures with rhodamine dextran to confirm that the structures can act as functional vessels. Study in this direction will facilitate future reconstitution of vascularized human organs [19].

## Conclusions

**Figure 1:**
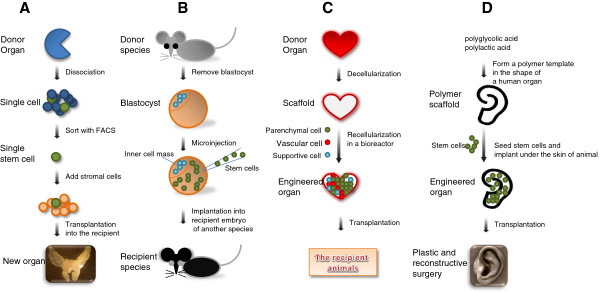
**Four approaches of generating functional organ. A,** From a single adult tissue stem cell to a functional organ. **B,** Generation of organs using blastocyst complementation system. **C,** Decellularization of matrix bioscaffolds and recellularization to produce a new organ. **D,** Combination of tissue-engineering and seeded stem cells to generate new organs.

In summary, as described above, there are four major ways of generation of functional organs using stem cells (Figure 1). Each of them has its own uniqueness. Specifically, using adult stem cells such as adult tissue epithelial stem cells, there will be a very low risk of tumorigenesis, more selective differentiation capability and better integration into the related tissue mass and acquisition of relevant organ functions. However, the number of the adult tissue stem cells that can be isolated is a limiting factor. Although using blastocyst complementation system is an attractive approach, particularly using a human-porcine inter-species strategy, injection of human stem cells into an organ-deficient porcine embryo is currently forbidden for ethical reasons. In fact, blastocyst complementation across species of large livestock animals such as porcine and canine has not been experimentally proven, more studies are needed in this direction. While the decellularization and recellularization research on various types of organs including the heart, liver, lung, kidney, bladder, etc. has yielded exciting results, but it is not practical to get the donor organs for such approach from the human beings. In addition, the acquisition of the organ functions from these studies is still limited. Tissue engineering using various biomaterial scaffolds holds hopes for particular medical needs such as plastic and reconstructive surgery, however the scaffolds using particular biomaterials are still primitive and require further development and research. Finally, the stem cell types that can be considered to be used for generation of functional organs may include ESC, iPSC and adult tissue cells. Each of these cell types has different advantages and disadvantages, in terms of proliferation, differentiation and tumorigenicity risk, which have been well-discussed in the literature.

Although organogenesis is a complex process, the organ generation systems described above using stem cells or a combination of stem cells and tissue engineering may be applied, or at least raise the hope, to treat organ failure in humans in the near future. Besides adult tissue stem cells and embryonic stem cells, recent burgeoning and promising development of technology of induced pluripotent stem cells opens a new avenue for potential cell replacement and organ generation. Relevant to generation of functional organs, it is worth-mentioning that functional hepatocyte-like cells can be generated from induced pluripotent stem cells and the liver can be partially reconstituted in Fah−/− mice [20].

Taken together, with the above initial steps and further improvement, stem cell therapy may one day not only repair tissue damage but also generate new tissues for tissue/organ transplantation. Even though it is still at an infancy stage, these studies may hold promise for generation of specific functional organs for organ transplantation, to help solve the clinical problem of donor shortage.
